# Readon: a novel algorithm to identify read-through transcripts with long-read sequencing data

**DOI:** 10.1093/bioinformatics/btae336

**Published:** 2024-05-28

**Authors:** Siang Chen, Hao Wang, Dongdong Zhang, Runsheng Chen, Jianjun Luo

**Affiliations:** Key Laboratory of Epigenetic Regulation and Intervention, Institute of Biophysics, Chinese Academy of Sciences, Beijing 100101, China; College of Life Sciences, University of Chinese Academy of Sciences, Beijing 100049, China; Key Laboratory of Epigenetic Regulation and Intervention, Institute of Biophysics, Chinese Academy of Sciences, Beijing 100101, China; College of Life Sciences, University of Chinese Academy of Sciences, Beijing 100049, China; Key Laboratory of Epigenetic Regulation and Intervention, Institute of Biophysics, Chinese Academy of Sciences, Beijing 100101, China; Key Laboratory of Epigenetic Regulation and Intervention, Institute of Biophysics, Chinese Academy of Sciences, Beijing 100101, China; College of Life Sciences, University of Chinese Academy of Sciences, Beijing 100049, China; Key Laboratory of Epigenetic Regulation and Intervention, Institute of Biophysics, Chinese Academy of Sciences, Beijing 100101, China; College of Life Sciences, University of Chinese Academy of Sciences, Beijing 100049, China

## Abstract

**Motivation:**

There are many clustered transcriptionally active regions in the human genome, in which the transcription complex cannot immediately terminate transcription at the upstream gene termination site, but instead continues to transcribe intergenic regions and downstream genes, resulting in read-through transcripts. Several studies have demonstrated the regulatory roles of read-through transcripts in tumorigenesis and development. However, limited by the read length of next-generation sequencing, discovery of read-through transcripts has been slow. For long but also erroneous third-generation sequencing data, this study developed a novel minimizer sketch algorithm to accurately and quickly identify read-through transcripts.

**Results:**

Readon initially splits the reference sequence into distinct active regions. It employs a sliding window approach within each region, calculates minimizers, and constructs the specialized structured arrays for query indexing. Following initial alignment anchor screening of candidate read-through transcripts, further confirmation steps are executed. Comparative assessments against existing software reveal Readon's superior performance on both simulated and validated real data. Additionally, two downstream tools are provided: one for predicting whether a read-through transcript is likely to undergo nonsense-mediated decay or encodes a protein, and another for visualizing splicing patterns.

**Availability and implementation:**

Readon is freely available on GitHub (https://github.com/Bulabula45/Readon).

## 1 Introduction

Unlike prokaryotes where polycistronic transcription is common, the traditional view in the human genome is that each gene is transcribed independently. However, although transcription typically terminates at a defined termination site, there are instances where the upstream termination signal is ignored. This leads to the transcription of intergenic regions, continuing into the next gene and resulting in the formation of a read-through fusion transcript. The necessary conditions for the formation of read-through transcripts involve active transcription of upstream genes and transcriptional detour at transcription boundaries (Barresi *et al.* 2019).

The subsequent fate of the read-through transcripts, whether they are translated into proteins or undergo degradation, remains uncertain. It is also critical to determine whether this is a random or controlled process. Based on the splicing pattern of the intergenic region, analyzing whether a premature stop codon (PTC) is introduced will help predict whether the transcript is likely to be degraded through the nonsense-mediated decay (NMD) pathway (Hug *et al.* 2016) or has the potential to be translated into a protein. Read-through transcription that do not generate fusion transcripts but do enter the 3ʹ untranslated region (3ʹ UTR) tend to produce aberrant proteins, which can be targeted and cleared by a coupled, two-level quality control pathway involving the BAG6 chaperone complex and the ribosome-collision-sensing protein GCN1 (Müller *et al.* 2023). There is a prevailing consensus that read-through of fusion transcripts is generally considered to be a controlled process, as evidenced by their unique expression patterns that are also observed in various cancer cell lines. For example, SLC45A3-ELK4, SLC2A11-MIF, and RRM2-C2orf48 were identified to exhibit specific high expression in prostate cancer, cervical cancer, and nasopharyngeal carcinoma cell lines, respectively (Rickman *et al.* 2009, Han *et al.* 2017, Wu *et al.* 2018). Numerous studies have also confirmed the involvement of many read-through transcripts in the initiation and progression of tumors. For instance, KDSR-BCL2 read-through transcript induces the upregulation of the downstream oncogene *BCL2* in kidney cancer (Grosso *et al.* 2015). TWE-PRIL, which encodes a bifunctional protein, stimulates cycling in T- and B-lymphoma cell lines (Kolfschoten *et al.* 2003). Additionally, CTSD-IFITM10 plays a role in breast cancer cell proliferation (Varley *et al.* 2014).

However, in the era of next-generation sequencing, detecting read-through transcripts poses significant challenges. With traditional Illumina-based methods, such as DEEPEST (Dehghannasiri *et al.* 2019) and Fusion-Bloom (Chiu *et al.* 2020), since the reads are individual short fragments, standard processing pipelines involving alignment and annotation tend to naturally separate these fragments, counting them independently as upstream or downstream gene fragments. One potential solution is *de novo* assembly, but it is known that *de novo* assembly of sequencing data has been a challenging task, involving a delicate balance of accuracy and completeness, as well as the difficulty of assessing assembly quality (Baker 2012).

With the advent of third-generation sequencing data, we now have access to full-length transcript information, enabling natural retrieval of entire transcript sequences. The key lies in recognizing that the read-through transcript is composed of two segments from the upstream and downstream genes. Previous related works, such as Genion (Karaoglanoglu *et al.* 2022), JAFFAL (Davidson *et al.* 2022), and LongGF (Liu *et al.* 2020), have addressed the problem of identifying fusion transcripts from third-generation sequencing data. However, the fusion events detected by Genion arise primarily from structural genomic variation. JAFFAL can recognize various fusions, but it is a packaged workflow that requires preparation of software tools such as Minimap2 (Li 2018), blat (Kent 2002), Oases (Schulz *et al.* 2012), and Velvet (Zerbino and Birney 2008). Therefore, results can be affected by various combinations of parameters of these tools. LongGF, on the other hand, as a downstream post-processing tool, requires to input data generated after alignment with Minimap2 or other similar tools. It is worth noting that typical aligners only output secondary alignments only under certain conditions. For example, in minimap2, when two alignments overlap, the shorter one is considered a secondary alignment and output only if its score exceeds 0.8. However, for read-through transcripts, which are combinations of adjacent genes, it is easy for reads from one gene to be considered a set of primary and secondary alignments. Therefore, a very low score threshold needs to be set here. While allowing such secondary alignment will significantly increase computation time and resulting file size, complicating downstream processing. Furthermore, JAFFAL identifies fusion transcripts by considering whether there is a certain quantity of supporting reads near the fusion site. While this approach still continues the idea of identifying fusion sites from next-generation sequencing data, it may not be suitable for third-generation sequencing data, which generally have lower sequencing depth and higher error rates.

To address this issue, we developed a novel minimizer sketch algorithm (named Readon). Readon effectively utilizes the neighboring position information of upstream and downstream genes by isolating the genome into distinct active regions. It employs a sliding window within each region, calculates the minimizer and builds a specialized, query-efficient, memory-efficient data structure to store minimizers. This enables rapid screening of numerous sequences that are less likely to be detected as read-through transcripts at early stages, followed by a more thorough validation on the smaller set of candidates. Readon can quickly and accurately identify read-through transcripts. When evaluated on simulated and validated real data, Readon demonstrated higher sensitivity than previous methods. Analysis of single-cell data further showed that the read-through transcripts identified by Readon were able to capture some of the differences between cell populations.

Additionally, Readon is a standalone software developed from scratch, eliminating dependencies on other software installations and simplifying configuration for user-friendly operation. Given the limited research on read-through transcripts, we also provide two downstream analysis tools: (i) predicting the fate of read-through transcripts by internally correcting inserted intergenic regions and checking for the presence of PTCs. (ii) Visualizing the alignment of read-through sequences to facilitate in-depth analysis of splicing patterns.

## 2 Materials and methods

**Figure 1.**
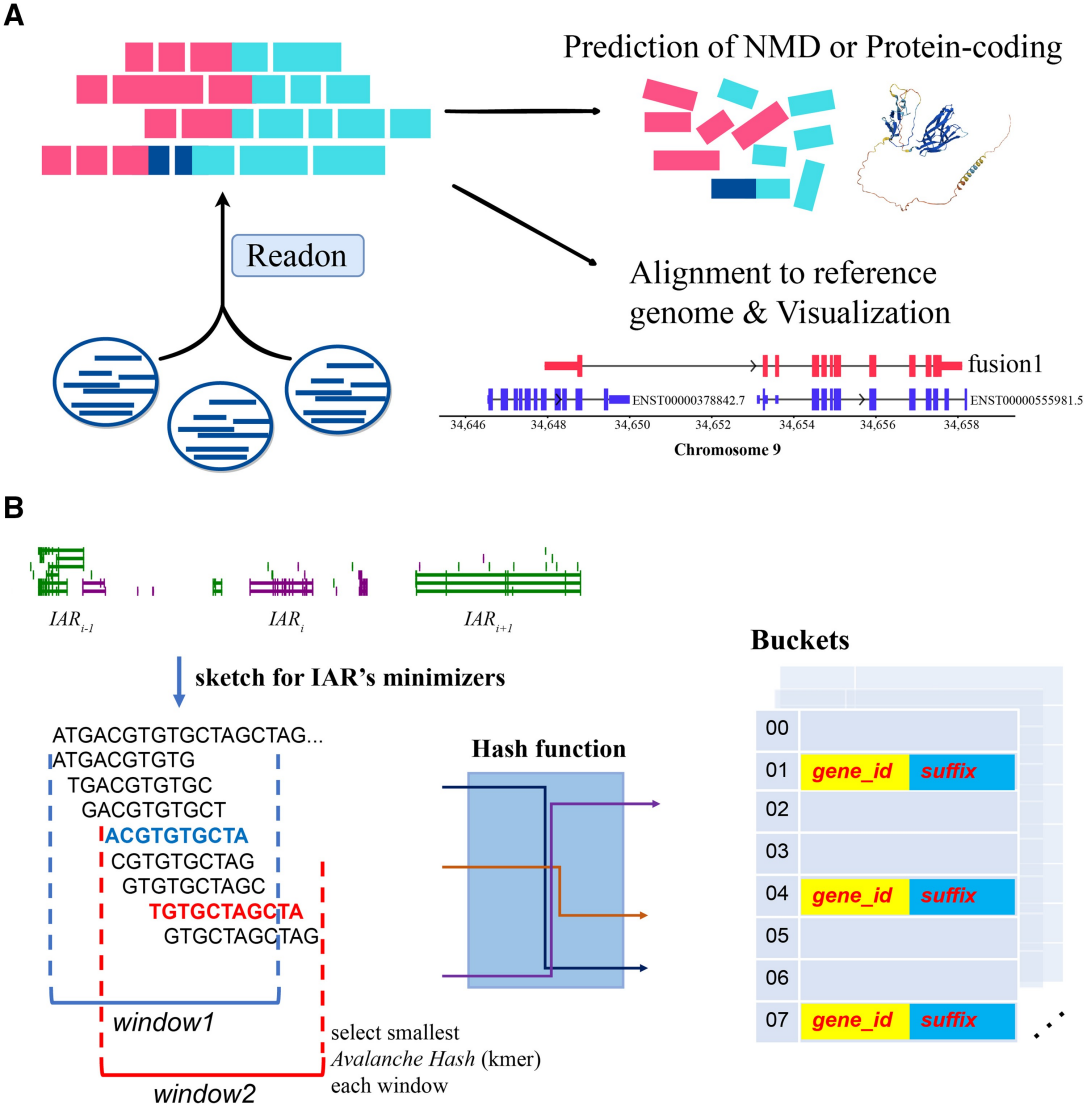
A schematic illustration of the Readon application and algorithm. (A) Readon identifies read-through transcripts from long-read sequencing data. Two downstream tools are also provided: one for predicting whether a read-through transcript is likely to undergo NMD or encode a protein, and another for visualizing splicing patterns. (B) First, order the genes on the genome according to the 5ʹ start position of the genes, with consecutive multiple genes constituting an isolated active region (IAR). Second, apply a sliding window of fixed length to genes in each IAR, and use a unique mapping avalanche hash function to calculate minimizer of the window. Then, an index table is constructed for each of these regions for rapid localization. Once potential candidates were identified, it can be further confirmed whether hit minimizers of sequences in the candidate regions originate from upstream or downstream genes.

Considering that read-through transcripts are composed of adjacent upstream and downstream genes, and that the vast majority of reads in sequencing data are transcribed from a single gene, we aimed to leverage this characteristic from the outset. Thus, we first ordered the genes on the genome according to the 5ʹ start position of the genes, with consecutive multiple genes constituting an isolated active region (IAR), and between the IARs are desert regions with no genes. Then, an index table is constructed for each of these regions for rapid localization. Once potential candidates were identified, the hitting minimizers of the sequences in the candidate regions are further confirmed whether they are from the upstream and downstream genes. By comparing hit counts, the most likely candidates are output, and users can adjust the number of supported read filters. Figure 1A summarizes the analysis performed herein using Readon and its applications. Figure 1B describes the algorithmic schematic of Readon.

### 2.1 Dividing isolated active regions

Let G represent a reference genome, and $\mathrm{gen}e_{1},\mathrm{gen}e_{2},\ldots ,\mathrm{gen}e_{N}$ denote genes in G, where N is the total number of genes, and $\mathrm{star}t_{i}<\mathrm{star}t_{i+1}\;\text{for}\;i\in 1,2,\ldots ,N$. Then, we set a distance threshold thr and consecutively examine the intergenic distances. If $\mathrm{star}t_{i+1}-end_{i}>\mathrm{thr}$, $\mathrm{gen}e_{i}$ and $\mathrm{gen}e_{i+1}$ will be added to different IAR buckets; otherwise, they will be added to the same IAR bucket. After this process, we obtain $\mathrm{IA}R_{1},\mathrm{IA}R_{2},\ldots ,\mathrm{IA}R_{j},\ldots ,\mathrm{IA}R_{M}$, with a total of M IARs. In $IAR_{k}$, there are $n_{j}$ genes $\mathrm{gen}e_{j,1},\mathrm{gen}e_{j,2},\ldots ,\mathrm{gen}e_{j,n_{j}}$, such that $\mathrm{star}t_{j,1}-end_{j-1,n_{j-1}}>\mathrm{thr}$ and $\mathrm{star}t_{j+1,1}-end_{j,n_{j}}>\mathrm{thr}$.

### 2.2 Sketching for IAR’s minimizers

Next, to consider the processing of a specific $IAR_{j}$, and the others are similar, we apply a sliding window of length s with a step size of st to $\mathrm{gen}e_{j,l}$ for $l\in 1,2,\ldots ,n_{j}$ with length $L_{j,l}$. In each window, there are s-k+1 consecutive kmer sequences. We utilize a unique mapping avalanche hash function to calculate the hash value of each k-mer, select the minimizer as the k-mer corresponding to the minimum hash value, and store it. The concept of the minimizer was proposed by minimap2 (Li 2018), providing with improved compression (Jain *et al.* 2020). The minimizer selection is determined by the following equation:

$$\mathrm{minimizer}=\underset{i\in \{1,2,\ldots ,s-k+1\}}{\text{argmin}}hash\left(\mathrm{kmer}\right)$$

### 2.3 Indexing in a specialized structured array

In addition, given the large scale of the reference sequence and sequencing files, there are numerous memory reads during sequence alignment process. Many built-in hash table data structures in programming languages, such as unordered set in C++ and dictionaries in Python, serve as black boxes that encapsulate various complex methods, memory checks, and resizing mechanisms for diverse user applications. Particularly, these structures are stored using linked lists when hash collisions occur, which is computationally intensive for genome-scale sequences. Therefore, this study addresses this specific issue by redesigning an array-based hash table implementation, which proves to be very efficient for frequent read operations due to its contiguous memory.

Considering the need to store minimizers $mmz_{1},mmz_{2},\ldots ,mmz_{MI_{j}}$, for $IAR_{j}$, where the total number of minimizers is $MI_{j}$, we introduce a parameter mo. The index of $mmz_{i}$ in the array is given by:

idxi=hashmmzi % MIj, MIj≥mohashmmzi % mo, MIj<mo

$$for\;any\;i\in \{1,2,\ldots ,MI_{j}\}.$$

Through the above approach, we have established a hash table with a specific size for each IAR bucket. This not only conserves memory and enables fast mappings but also avoids missing alignments. We have demonstrated this in the supplementary materials.

### 2.4 Prediction of NMD or protein-coding and visualization

**Figure 2.**
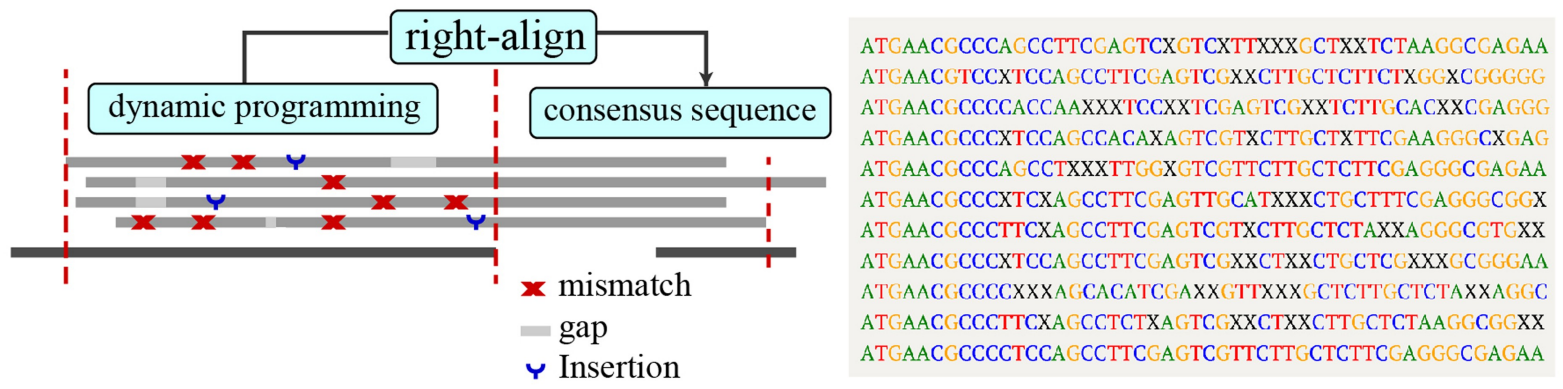
A schematic illustration of the Readon application and algorithm. Align multiple sequences to the start site of the upstream gene until the boundary exon is reached. Then, reciprocal correction of intergenic segments was performed to identify consensus sequences. Finally, the distance from the stop codon (UAA, UAG, and UGA) to the downstream exon-exon junction is obtained.

The NMD pathway is one of the cellular regulatory mechanisms for detecting and eliminating defective gene products. During normal translation, ribosomes scan the last exon-exon junction and displace the exon junction complex (EJC), continuing to scan until a stop codon is reached. In the case of a premature termination codon due to a nonsense mutation, the exon-exon junction downstream of the premature stop codon still has an undisplaced EJCs. This situation triggers NMD. However, if the nonsense mutation occurs within 55 nucleotides of the last exon-exon junction, the ribosome can displace the EJC before it reaches the premature termination codon, thus preventing NMD. This phenomenon is encapsulated by the “55nt rule” of the EJC model (Brogna and Wen 2009). In our prediction model, we first align multiple sequences to the start position of the upstream gene, aligning them with the reference sequence until reaching the boundary exon. Subsequently, we performed reciprocal correction of intergenic segments, identified consensus sequences, and ultimately assessed whether the premature termination codon was located within 55 nucleotides of the last exon-exon junction, as shown in Fig. 2.

To facilitate a comprehensive examination of read-through transcripts for splicing patterns, including exon combinations in upstream and downstream genes, as well as insertions into intergenic regions, we provide a visualization script. It initially extracts the sequence from the transcription start site of the upstream gene to the transcription termination site of the downstream gene. Then, it conducts a sequence alignment query on the reference sequence, producing alignment results in BED format. BED files of upstream and downstream genes were concurrently used as inputs for graphical visualization.

### 2.5 Data resources

Human annotations used in our results are from Ensembl release 110 (ftp.ensembl.org/pub/release-110/gtf/homo_sapiens/). All sequencing data used in this study is publicly available, as detailed in Supplementary Table S1.

## 3 Results

Readon is implemented in C/C++, compiled and executed on Linux systems, and supports input in both fasta and fastq formats. Utilizing annotated information in the Ensembl database, we systematically investigated the sequence and expression characteristics of read-through transcripts. Performance comparison of Readon with other methods on simulated and real data reveal that Readon exhibits the highest recall and precision. Application to single-cell third-generation sequencing data also demonstrated the distinguishability of read-through transcripts identified by Readon across cell types. We measured the memory usage and runtime of Readon and existing tools on datasets of different sizes, as shown in Table 1. Readon demonstrates lower memory consumption and runs faster.

**Table 1.** Comparison of runtime and memory usage across datasets of different sizes.^a^

	Tool	Memory (GB)		Runtime (min)	
		μ	σ	μ	σ
2.8 billion reads	JAFFAL	10.29	0.014	48.54	2.18
	LongGF	11.41	0.11	27.18	1.52
	Readon	3.71	0.00	15.67	1.50
4.9 billion reads	JAFFAL	10.43	0.00	443.74	25.17
	LongGF	10.29	0.014	162.51	4.81
	Readon	4.41	0.00	48.91	3.14

a The table above displays the average and standard deviation of runtime and memory usage across three runs for each dataset.

### 3.1 Analysis on annotated read-through transcripts

**Figure 3.**
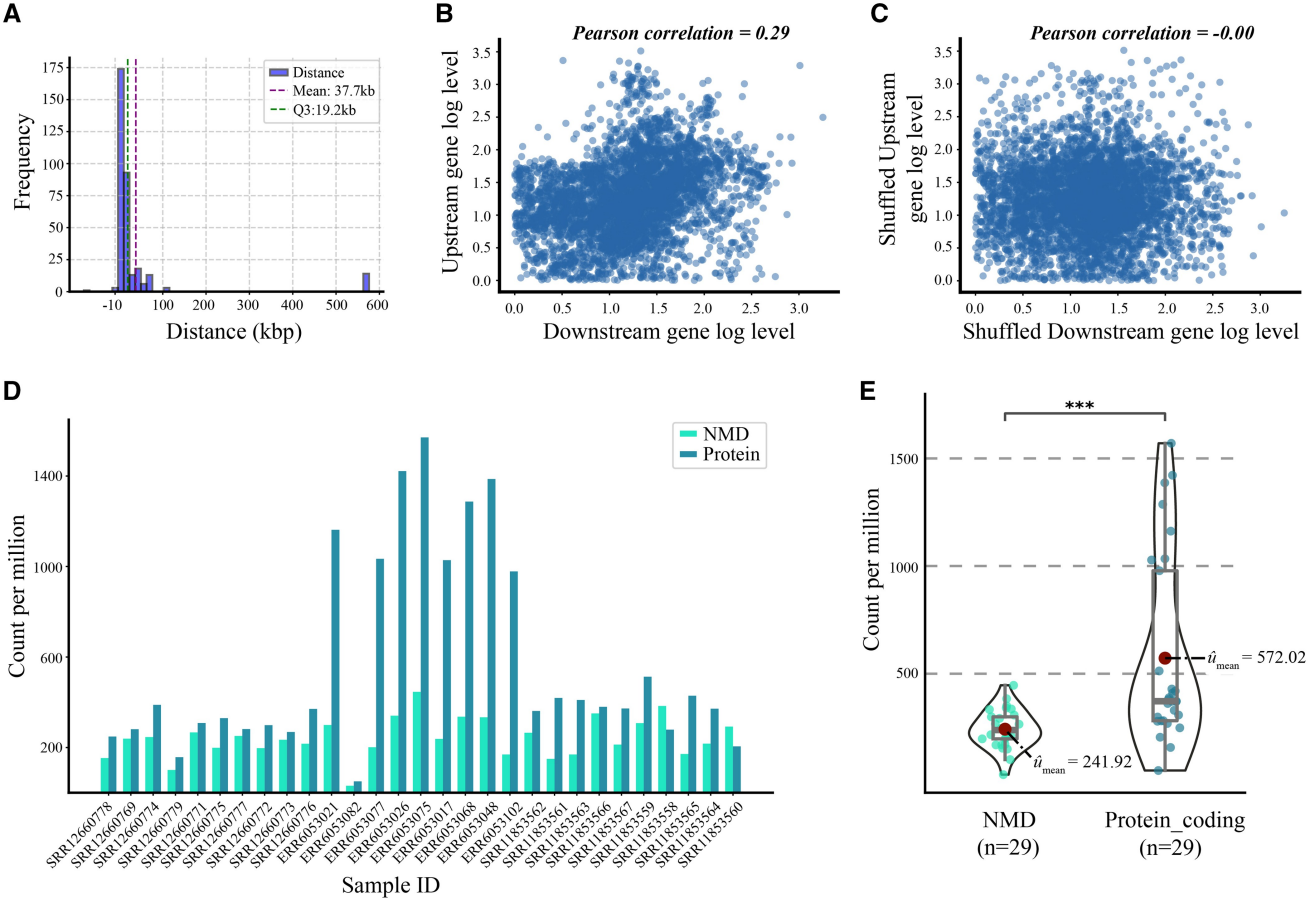
Analysis on annotated read-through transcripts. (A) The distribution of distances between the transcription termination sites of upstream genes and the transcription start sites of downstream genes of annotated read-through transcripts. (B) and (C) The correlations of paired and randomly shuffled expression levels of upstream and downstream genes across different tissues. (D) The histogram plot of Counts per Million (CPM) for NMD versus protein-coding types. (E) The violin plot of CPM for NMD versus protein-coding types, and *P*-value reported from a t test is < 0.001.

Figure 3A displays the distribution of distances between the transcription termination sites of upstream genes and the transcription start sites of downstream genes for annotated read-through transcripts. The vast majority of distances are below 100 kbp, primarily due to the presence of relatively strong transcription termination signals within a certain length of the genomic region, causing the transcription complex to halt. This length distribution can also provide a reference for setting distance threshold in Readon. However, there are exceptions where distances exceed expectations, as seen in IQCJ-SCHIP1. It contains 11 exons from upstream and downstream genes and spans 828 kb of genomic DNA (Kwaśnicka-Crawford *et al.* 2006), encoding a protein potentially contributing to axon initial segment maintenance (Papandreou *et al.* 2015).

However, even though such short distances between genes may indicate co-transcription, this does not imply that the expression of the downstream gene is necessarily dependent on the promoter of the upstream gene. This holds true for only a few genes in cancer cells, such as the oncogene BCL2, where BCL2 mRNA and protein levels correlated positively with the levels of transcription read-through of the KDSR gene located immediately upstream (Grosso *et al.* 2015). According to the normal tissue expression data downloaded from GTEx, there are no significant differences in the expression of upstream and downstream genes, indicating that the promoters of downstream genes are sufficiently utilized (Supplementary Fig. S1). On the other hand, considering the paired expression patterns of upstream and downstream genes across various tissues, as depicted in Fig. 3B and C, there is still a weak correlation in their expression. This can be understood as that they belong to the same chromosomal activity cluster while exhibiting relatively high or low expression. This indirectly supports our rationale for choosing the IAR approach.

Subsequently, we annotated these read-through transcripts on publicly available third-generation sequencing data. Figure 3D and E depict Counts per Million (CPM) for NMD and protein-coding types. Remarkably, the CPM for protein-coding read-through transcripts is significantly higher than that of NMD transcripts. This observation primarily stems from the fact that transcriptome sequencing captures a transient snapshot of cellular expression, and the shorter half-life of NMD-type transcripts results in lower detectable quantities.

Overall, the above analysis provides an examination of previously discovered full-length transcripts, exploring the distribution of intergenic distances and expression patterns of these transcripts. This helps us gain a more systematic understanding of read-through transcripts.

### 3.2 Performance on simulated data and parameter optimization

Since read-through transcripts have been largely overlooked in the past due to limitations of next-generation sequencing or discarded during data processing, their distribution remains unknown. Therefore, we employed Nanosim (version 2.5.0) to generate datasets and it can simulate technology-specific features of ONT data, such as sequencing fragment length coverage, and sequencing error rate (Yang *et al.* 2017). Nanosim's model is trained on public ONT R10.4 data, which is available from the sequence read archive (SRA) under accession SRP395331 (Ni *et al.* 2023), and the reference transcriptome includes cDNA sequences of individual gene transcripts and sequences generated by splicing two adjacent genes. For normal transcripts and read-through transcripts, we assigned equal expression levels. On average, 949 transcripts, consisting of regular transcripts and simulated read-through transcripts, were randomly selected and generated across all chromosomes. The number of reads ranged between 90 000 and 100 000, ensuring a sequencing depth of ×100.

**Figure 4.**
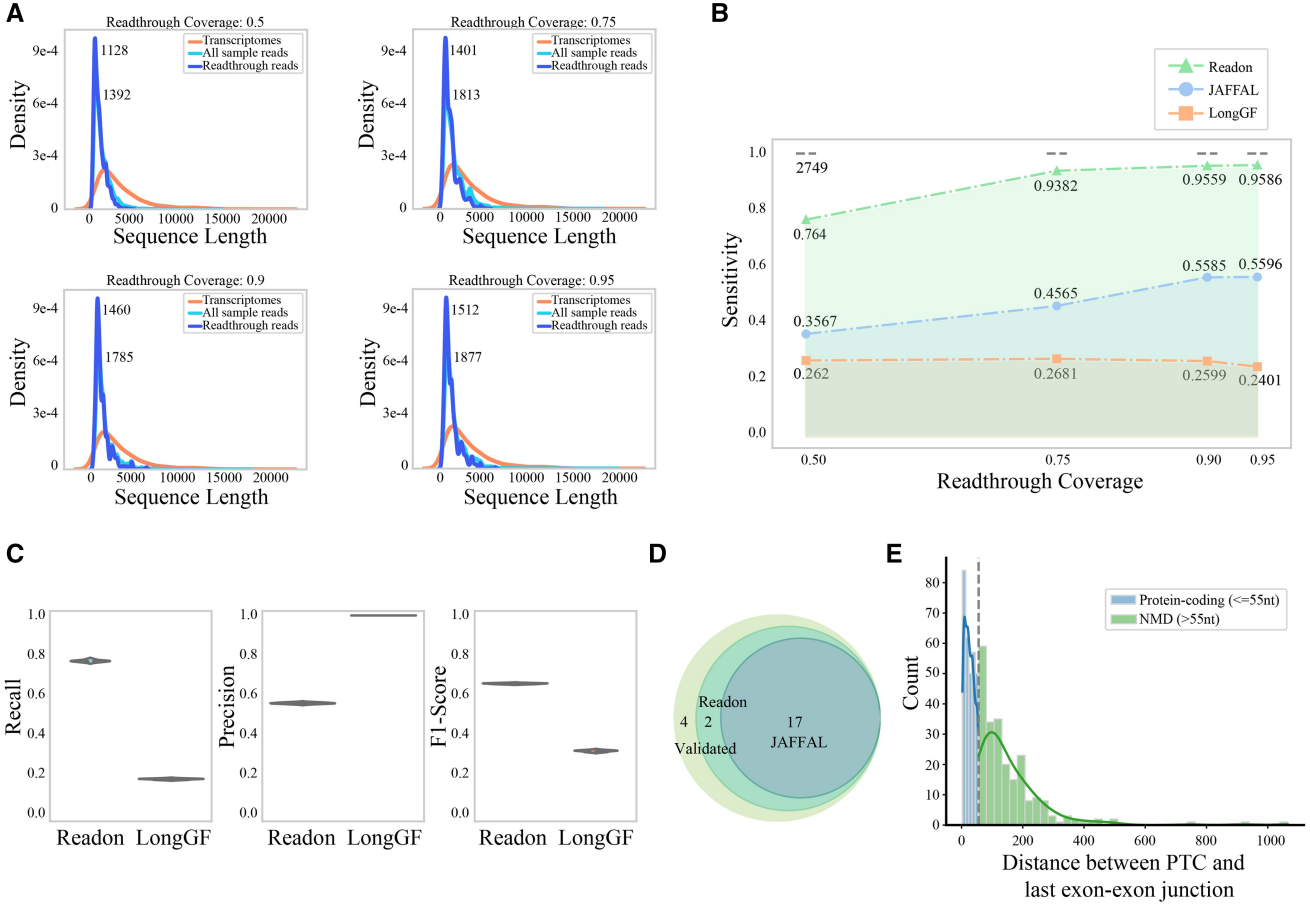
Assessments on simulated data and validated real data. (A) The length distribution of transcriptomes, all sampled reads and read-through reads under different read coverages (0.5, 0.75, 0.9, 0.95). (B) The sensitivity under different read coverages of Readon, JAFFAL, and LongGF in high-quality simulated data. (C) Recall, Precision, and F1-score of Readon versus LongGF on detecting read-throughs from erroneous simulated data. (D) Venn plot of Readon and JAFFAL on detecting validated read-throughs in cancer cell lines. (E) The distribution of distances between the stop codon sites and the downstream last exon–exon junctions of read-throughs.

Initially, we simulated the generation of high-quality sequencing data by setting the—perfect option, which means it would ignore error profiles in the training model. To ensure fairness, we compared the performance of Readon, JAFFAL, and LongGF under different read coverages (0.5, 0.75, 0.9, 0.95) using default parameters. Coverage values refer to the minimum of Readon, JAFFAL, and LongGF under different read coverages (0.5, 0.75, 0.9, 0.95) using default parameters. Coverage values refer to the minimum length coverage per read; thus, only reads with coverage greater than that length will be simulated. We set different length coverage thresholds to test the performance of different software under conditions of high or low sequence integrity. Figure 4A presents the length distribution of the sampled reads under different read coverage parameters. An increase in read coverage implies that the sampled sequences more closely resemble the original full-length transcripts. As shown in Fig. 4B, Readon and JAFFAL identify more read-throughs with increasing read coverage, while LongGF consistently detects few read-throughs. Overall, Readon achieves the best sensitivity under different coverage settings. The precision of all three tools are over 0.95 as shown in Supplementary Fig. S2A.

Given the diversity of variants in real sequencing samples, we introduced simulated data with mismatches, insertions, deletions, and sequencing errors on the reference sequence. JAFFAL identified very few read-through transcripts, typically in single digits. This limitation is attributed to JAFFAL's stringent requirements for high precision and depth near splicing sites, making it less suitable for data with errors and mutations. Our comparison focused on Readon and LongGF, LongGF's recall is approximately 0.2, while Readon consistently achieves a recall exceeding 0.8 and has a higher F1-score (Fig. 4C). All tests above were performed using Readon's default parameters (k-mer length is 25 and window size is 40). We also tested its performance with smaller k-mer length, and the results are presented in Supplementary Fig. S2B.

### 3.3 Detection of validated read-throughs in cancer cell lines

We applied Readon to third-generation sequencing data (Supplementary Table S1) from the human breast cancer cell line MCF-7, specifically selected for its well-established fusion transcripts, and we set k-mer length to 15, the window size to 24, and the sliding step to 9. Leveraging precompiled tables (Davidson *et al.* 2022), we investigated gene pairs' chromosome and position details, focusing on potential read-through transcripts between adjacent genes (Supplementary Table S2). A total of 23 instances were identified. As shown in Fig. 4D, in the results provided by Davidson *et al.* (Davidson *et al.* 2022), JAFFAL failed to detect six instances. Readon missed only four. Notably, Readon relies exclusively on third-generation sequencing data, emphasizing our aim to leverage long-read sequencing, despite its inherent error rate, for identifying read-through transcripts, without the need for additional high-quality next-generation data. Incorporating extra data would entail additional costs. Further examination of the four undetected read-throughs revealed their absence in the third-generation sequencing files, suggesting insufficient expression levels as a potential cause for nondetection.

### 3.4 Downstream tools and analysis on human single cells

Taking a set of MCF-7 data from SGNex as an example, we applied our prediction tool to identified read-through transcripts. After computing the consistent sequences in the inserted region, we analyzed the distance between the stop codon site and the last exon–exon junction. Figure 4E illustrates the distribution of these distances, revealing that a majority of sequences introduce premature termination codons (PTCs). These sequences are likely subjected to degradation and might lose their functionality. However, a small subset of sequences lacks PTCs, suggesting potential translation into proteins. These proteins could exhibit dual functionality, similar to the separate translation of the original two genes. Alternatively, they might possess unpredictable new functions or activate downstream genes that were initially not expressed or expressed at low levels. In summary, these findings indicate a significant impact on cellular metabolism.

Additionally, in Supplementary Fig. S3, we present examples of two read-through transcripts to demonstrate how our analysis tool visualizes splicing patterns. In Supplementary Fig. S4, we utilized the expression matrix of identified read-through transcripts for dimensionality reduction and clustering in single-cell third-generation sequencing data. It demonstrates that read-through transcripts can partially represent differences between cell populations, although not prominently. We speculate that this is due to the insufficient ability of read-through transcripts to distinguish cell types and the relatively low abundance and diversity of read-through transcripts in normal tissue single-cell third-generation data.

## 4 Discussion

Many selectively fused genes associated with each cancer have already helped us discover biomarkers and therapeutic targets, and read-through induced fusions represent a novel addition to this landscape (Vellichirammal *et al.* 2021, Dorney *et al.* 2023). Past mis-annotations may have been introduced by next-generation sequencing data, and long-read sequencing now holds significant promise for uncovering read-through transcripts.

To efficiently and accurately identify read-through transcripts from both high-quality and error-prone long reads, we divide the genome into multiple isolated active regions (IARs) for aligning. This approach helps pre-filter a significant number of noncandidates. During testing, we observed that the noncontiguous storage of hash tables in memory posed a computational bottleneck when aligning sequences in large-scale genomic and sequencing datasets. To address this challenge, we implemented a customized array-based hash table for each IAR, enabling parallelization at this level. The resulting tool, Readon, demonstrated excellent performance on both simulated and real datasets, fast identifying known read-through transcripts. We have also optimized parameter combinations for reference, which can be directly applied by the user.

Currently, a comprehensive understanding of read-through transcripts remains under-developed. Questions surrounding the origin of read-through transcripts and the selective transcription and splicing of intergenic regions persist. The autonomous generation of read-through transcripts by cells, and how cells monitor this process, if unconscious, remain unclear. The association between high expression of read-through transcripts and the functional role in cancer cells is also an area of interest. In conclusion, we hope that Readon serves as a foundational tool for researchers investigating these questions.

## Supplementary Material

btae336_Supplementary_Data

## Data Availability

All sequencing data used in this study is publicly available, as detailed in Supplementary Table S1. The source code of Readon is freely available on GitHub at https://github.com/Bulabula45/Readon.
