# Supplementary material for: Readon: a novel algorithm to identify read-through transcripts with long-read sequencing data
Source: Bioinformatics. 2024 May 28;40(6):btae336. doi: 10.1093/bioinformatics/btae336 (PMC11162696; doi:10.1093/bioinformatics/btae336)
Supplement: btae336_Supplementary_Data [file btae336_supplementary_data.zip › r2_Supplementary_data.pdf]

Readon: a novel algorithm to identify read-through transcripts with long-read sequencing data (Supplementary Materials)

1 Figures

Supplementary Figure 1

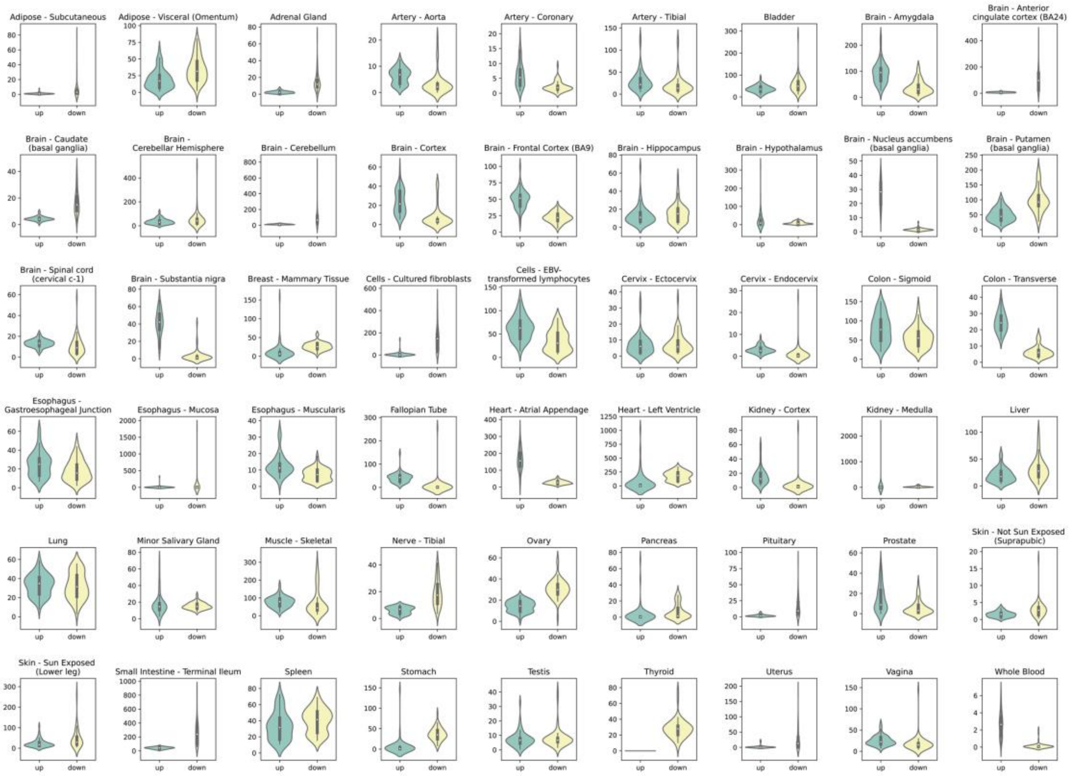

Fig. S1. A violin plot is used to depict the expression distribution of paired upstream and downstream genes in each normal tissue. In each plot, the left-sided violin represents upstream genes, and the right-sided violin represents downstream genes. The vertical axis corresponds to the gene expression Transcripts Per Million (TPM) values.

Supplementary Figure 2

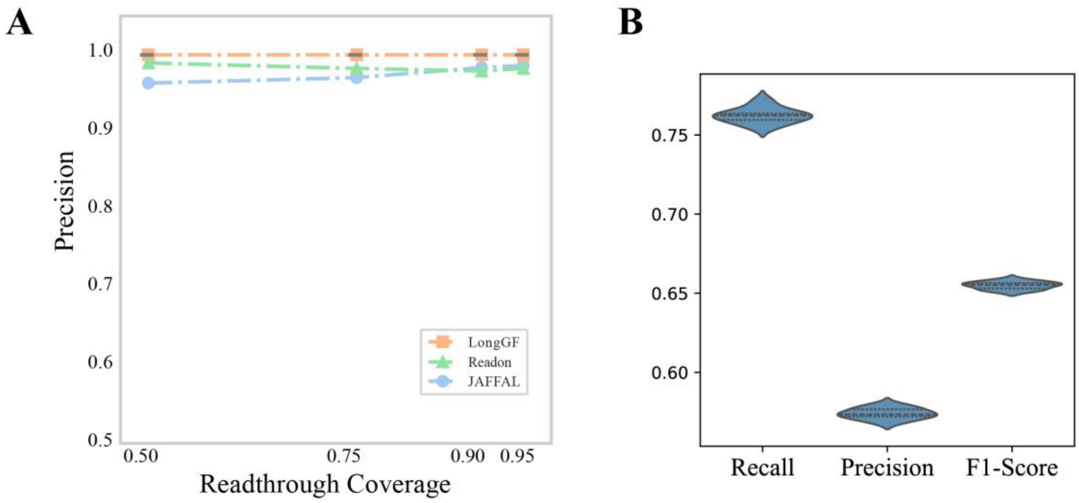

Fig. S2. Assessments on simulated data. (A) The precision under different read coverages of Readon, LongGF, and JAFFAL in high-quality simulated data. (B) Precision, recall and F1-score of Readon on detecting read-throughs from erroneous simulated data (k-mer length is 15 and window size is 24).

Supplementary Figure 3

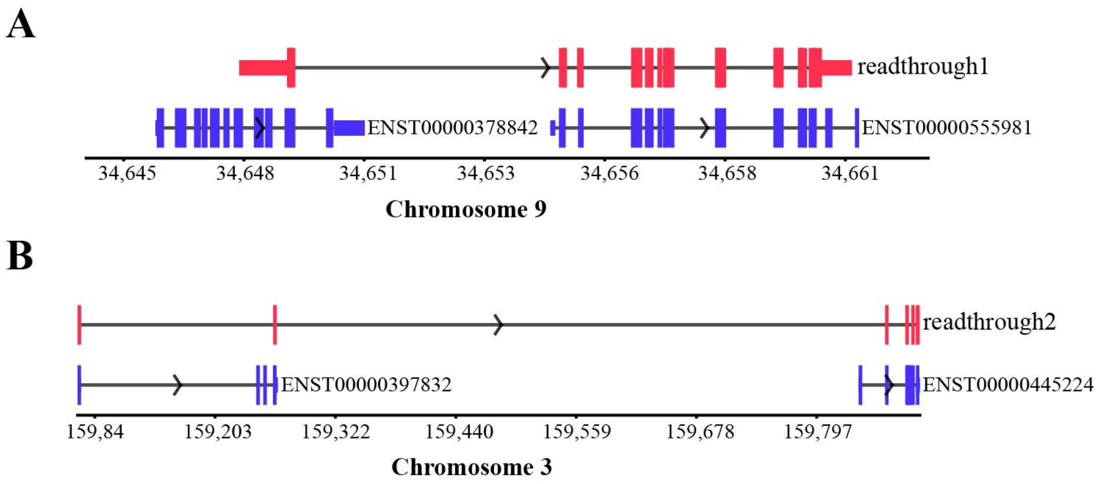

Fig. S3. The visualization of instances of readthrough transcript alignment to the reference. (A) An instance of read-through transcript in chromosome 9, which is composed of incomplete exons from the upstream gene and multiple exons from the downstream gene. (B) An instance of read-through transcript in chromosome 3, which is formed by the first and fourth exons from the upstream gene and exons beyond the first one from the downstream gene.

Supplementary Figure 4

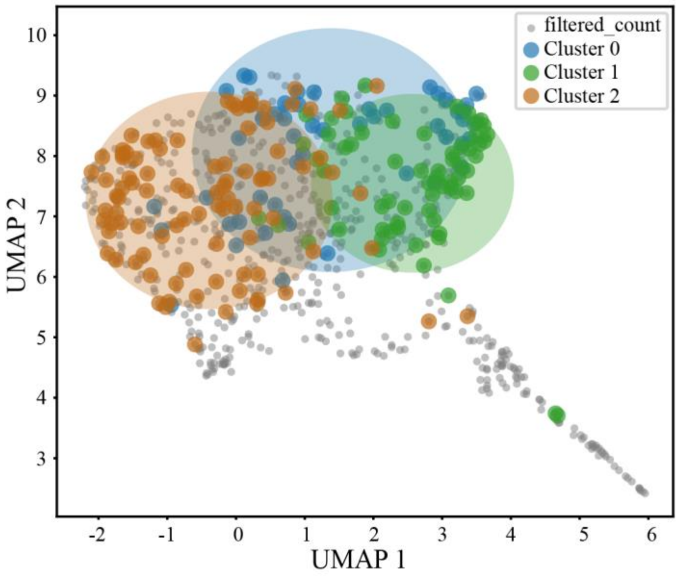

Fig. S4. UMAP plot of gene expression matrix of human single-cell PacBio sequencing of HEK293T cell line. Cluster 0, 1, and 2 are the labels obtained through dimensionality reduction and k-means clustering using the readthrough transcript expression matrix identified by Readon.

2 Details in algorithm

Here is the proof that readon's data structure can avoids missing alignments. First, some formulas from randomized algorithms will be introduced.

Theorem 1 (Markov's Inequality). *Let* $\boldsymbol{X}: S \to R$ *be a non-negative random variable. Then, for any* $a > 0$,

$$Pr(\mathrm{X} \geq \mathrm{a}) \leq \frac{\mathrm{E(X)}}{\mathrm{a}}$$

Theorem 2 (Chebyshev's Inequality). *Let* $X: S \to R$ *be a random variable with expectation* $\mathrm{E}(X)$ *and variance* $Var(X)$*. Then, for any* $a \in R$,

$$\mathrm{Pr}(|\mathrm{X} - \mathrm{E(X)}| \geq \mathrm{a}) \leq \frac{\mathrm{Var(X)}}{\mathrm{a}^2}$$

Proof. (of Markov's inequality.) According to the definition of mathematical expectation and the non-negativity of X,

$$\begin{aligned}
\mathrm{E(X)} &= \sum_{\mathrm{x} \geq 0} \mathrm{x\,Pr(X = x)} \\
&= \sum_{0 \leq \mathrm{x} < \mathrm{a}} \mathrm{x\,Pr(X = x)} + \sum_{\mathrm{x} \geq \mathrm{a}} \mathrm{x\,Pr(X = x)} \\
&\geq \sum_{\mathrm{x} \geq \mathrm{a}} \mathrm{x\,Pr(X = x)} \\
&\geq \sum_{x \geq a} a\,Pr(X = x) \\
&= \mathrm{aPr(X \geq a)}
\end{aligned}$$

Proof. (of Chebyshev's inequality.) Let $Y = \left(X - E(X)\right)^2$ *be a non-negative random variable.*

Applying the Markov's inequality to Y,

$$Pr(\mathbf{Y} \geq a^2) \leq \frac{E(\mathbf{Y})}{a^2} = \frac{Var(\mathbf{X})}{a^2}$$

Minimizers are selected through the mapping of Thomas Wang's integer hash function, therefore, we can assume that each kmer key within a bucket is independently and identically distributed (i.i.d.). The modulo operation of the total number of minimizers with respect to a 64-bit unsigned integer kmer key is utilized as the address stored in the hash table.

Considering a specific bin in IAR_j, there are MI_j independent binary random variables $X_1, X_2, \ldots, X_n$, where $p_i = \Pr(X_i = 1)$ and $X_i = 1$ corresponds to the case of the ith minimizer falling the bin. Let $X = \sum_i X_i$, and $E(X) = \mu$. For example, the load factor $\mu \approx 1/8$ when the distance threshold is $300\,kbp$. Then, we can apply the Chernoff bound to estimate the probability of minimizers hitting.

For any $\lambda > 0$, by Markov's inequality,

$$\Pr(X \geq (1+\delta)u) = \Pr\left(e^{\lambda X} \geq e^{\lambda(1+\delta)\mu}\right)$$
$$\leq \frac{E\left(e^{\lambda X}\right)}{e^{\lambda(1+\delta)\mu}}$$

Markov's inequality relies on first-order information, while Chebyshev's inequality incorporates second-order information. By employing a Taylor series expansion, Chernoff bound effectively incorporates information from all orders of the random variable.

Now, knowing that $X = \sum_i X_i$, we have,

$$\Pr(X \geq (1+\delta)u) \leq \frac{\prod_i E\left(e^{\lambda X_i}\right)}{e^{\lambda(1+\delta)\mu}}$$
$$= \frac{\prod_i \left(1 - p_i + p_i \cdot e^{\lambda}\right)}{e^{\lambda(1+\delta)\mu}}$$
$$= \frac{\prod_i \left(1 + p_i\left(e^{\lambda} - 1\right)\right)}{e^{\lambda(1+\delta)\mu}}$$
$$\leq \frac{\prod_i e^{p_i\left(e^{\lambda}-1\right)}}{e^{\lambda(1+\delta)\mu}}$$
$$= \frac{e^{\mu\left(e^{\lambda}-1\right)}}{e^{\lambda(1+\delta)\mu}}$$
$$= e^{\mu\left(e^{\lambda}-1-\lambda(1+\delta)\right)}$$

Taking the derivative of the above expression, we obtain its minimum value when $\lambda = ln(1+\delta)$,

$$\Pr(X \geq (1+\delta)\mu) \leq \left(\frac{e^{\delta}}{(1+\delta)^{1+\delta}}\right)^{\mu}$$

Let m denote the number of minimizer information stored in each hashtable's value. A hit occurs when there are $m+1$ or more minimizers in the same bin. If we use a 64-bit unsigned integer, we can store 4 values each key. Then, using $\mu = 1/8$, we have $\delta = (m+1)/\mu - 1 = 39$,

$$p = \Pr\left(X \geq (m+1)\right) \leq \left(\frac{e^{\delta}}{(1+\delta)^{1+\delta}}\right)^{\frac{1}{8}} \approx 1.28 \cdot 10^{-6}$$

Considering a transcript sequence s, let n_s denotes the number of minimizers, and k_s denotes the number of minimizers that hitted. The probability of having at least k minimizers all hit can be calculated as

$$\Pr(k_s \geq k) \leq 1 - \sum_{i=0}^{k-1} \binom{n}{i} p^i (1-p)^{n-i}$$

When $k = 5$, the probability of the bad event occurring is on the order of 10^{-22}, which is already more than sufficient for sequencing files of GB scale. In summary, this proves that our method of storing each IAR's minimizer as a hash table avoids missing alignments, while also saving memory and enabling fast mappings.
